# Current Molecular, Cellular and Genetic Aspects of Peri-Implantitis Disease: A Narrative Review

**DOI:** 10.3390/dj11050134

**Published:** 2023-05-16

**Authors:** Marek Chmielewski, Andrea Pilloni

**Affiliations:** 1 Private Practice, 80-280 Gdańsk, Poland; 2 Section of Periodontics, Department of Oral and Maxillo-Facial Sciences, Sapienza Unviersity of Rome, 00185 Rome, Italy

**Keywords:** inflammation, peri-implantitis, cellular response, molecular factors, genetic polymorphism, cytokines

## Abstract

(1) Background: Peri-implantitis is a multi-factorial disease with an inflammatory background that occurs in both soft and hard tissues surrounding implants. In recent years, the understanding of the cellular, molecular and genetic background of peri-implantitis has broadened. This study aims to summarize the currently available articles on the subject and highlight the most recent advances over the last 20 years. (2) Methods: For this study, the Embase and PubMed libraries were searched using the keywords: (“peri-implantitis” AND “cytokine” OR “genetics” OR “cellular”) and (“peri-implantitis” AND “cytokine” OR “genetics” OR “cellular” AND “risk factors”). The search revealed a total of 3013 articles (992 from PubMed, 2021 from Embase). Following screening of the titles and abstracts and full-text reads, 55 articles were included. (3) Results: In peri-implantitis IL-6, IL-1β, TNF-α, MMP-8 and their genetic variations appear to be the most important cytokines in relation to not only pathogenesis, but also their potential diagnostic capabilities. Epithelial and inflammatory cells, along with those of the bone lineage, are prime cellular elements found in peri-implantitis. (4) Conclusions: A wide array of cells stand behind peri-implantitis, as well as cytokines and their genetic variations that take part in the process. However, the growing interest in this topic has led to the introduction of specific new diagnostic tools to enable a better understanding of patients’ responses to treatment and, in turn, to even enable prediction of the risk of developing peri-implant disease.

## 1. Introduction

The wide spread of prosthetic restorations based on dental implants enables optimal oral rehabilitation of totally and partially edentulous patients, expanding the available treatment possibilities. Currently, the most common implant materials are pure titanium, Ti-6Al-4V alloy and zirconia. Additional modifications of the implant surface, for example acid etching and sandblasting or coating, enhance the osseointegration process, extend the bone–implant contact area and reduce the risk of implant failure [1]. The prevalence of dental implants in the global population is estimated to reach up to 23% by the year 2026 [2]. The growing number of patients translates to a higher number of potential peri-implant complications. One of these is peri-implantitis. It is defined as a progressive, irreversible disease affecting both hard (alveolar bone) and soft tissues (supracrestal tissues and mucosa) surrounding dental implants. The amount of keratinized mucosa, the supracrestal tissue height and the peri-implant bone thickness can all affect peri-implantitis occurrence [3]. Additionally, in peri-implantitis, there can be bone loss, hindered implant osseointegration and pathological pocket formation [4]. It is commonly associated with bacterial challenge regarding the inflammation of surrounding soft tissues and loss of bone support. The main bacteria responsible for the development of periodontitis belong to Socransky’s red complex. These are *Porphyromonas gingivalis, Tannarella forsythia* and *Treponema denticola* [5]. The peri-implant flora is similar, but the range of suspected bacteria involved in the development of peri-implant pockets is broader. The typical microbes found in the peri-implant pockets of peri-implantitis, which are often not specific, but commonly present, are *Campylobacter, Gemella, Bacteroides, Actinomyces, Peptostreptococcus, Streptococcus, Candida, Treponema, E. corrodes* and *P. nigrescens* [6]. While the microbial flora in peri-implantitis is relatively well known, much less information is available regarding the cellular and molecular responses. In medicine, cytokines are widely used as diagnostic and prognostic tools [7]. They are used to monitor the status of patients undergoing treatment for asthma, cancer, AIDS, heart disease, degenerative diseases and rheumatoid arthritis, to name only a few of them [7,8,9,10,11]. In dentistry, the use of cytokines is uncommon, only recently becoming more widespread [12]. One of the fields of dentistry that utilizes it is periodontics and implantology. An increase in implant surgeries and implant-supported restorations has led to a higher incidence of peri-implant disease occurrence in the population. The most-investigated biomarkers in periodontal and peri-implant tissues are L-1β, VEGF, MMP-8, TIMP-2 and OPG, as they alter soft and hard tissue cellular metabolism and appear in bacterial infections [13,14,15,16,17]. Peri-implant cervical fluid (PICF) is used as a site-specific and easy-to-obtain fluid. PICF is similar to gingival cervical fluid exerted from the gingival sulcus, and contains cells, bacteria, cytokines and active mediators [18]. To collect PICF, sterile paper strips are placed within the sulcus or peri-implant crevice and held for 30 s to properly soak up the fluid [19]. It is important that the paper strips do not become contaminated with blood or pus. The strips are then placed in tubes containing buffered saline and phenylmethylsulfonyl fluoride and centrifuged [19,20]. PICF is further used to conduct ELISA test for proteins and PCR for DNA and RNA, or to perform oral-based point-of-care (PoC) tests [21]. Currently, the ELISA test is the most widely used.

The primary aim of this study is to present an extensive review of the molecular, genetic and cellular factors affecting the course and intensity of peri-implantitis. The secondary aim is to highlight the potential of cytokines and their genetic polymorphism in early diagnostics and treatment prognosis.

## 2. Materials and Methods

**Figure 1:**
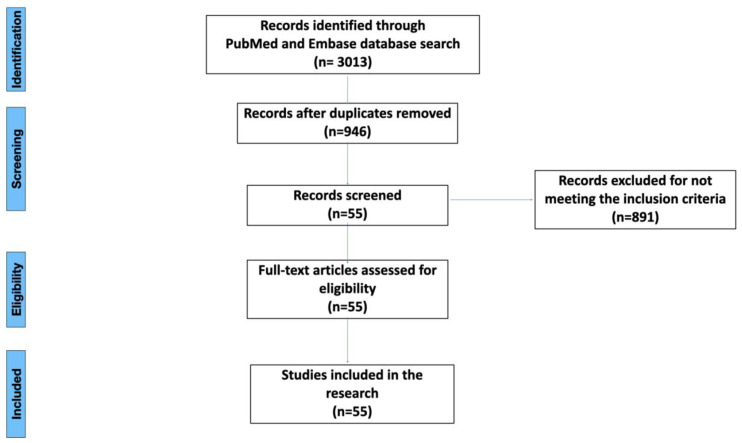
Flow chart for the inclusion and exclusion process.

A literature search of the Embase and PubMed databases was carried out form 30 June to 1 September 2022. The keywords used were: (“peri-implantitis” AND “cytokine” OR “genetics” OR “cellular”) and (“peri-implantitis” AND “cytokine” OR “genetics” OR “cellular” AND “risk factors”). The screening revealed 3013 articles in total: 992 from PubMed and 2021 from Embase. The selection process was conducted by two reviewers (MC and AP). During the initial search, duplicates were removed. After the initial search, title and abstract screening was performed in accordance with the inclusion criteria. The inclusion criteria were: (i) articles written in English, (ii) articles on molecular, genetic or cellular links to peri-implantitis, (iii) replicable study designs focusing on molecular, genetic or cellular aspects, (iv) studies carried out only on humans or grafted human tissues, and (v) studies that highlight the potential of cytokines in the pathogenesis, diagnosis or treatment of peri-implantitis. The exclusion criteria were: (i) studies published in languages other than English, (ii) studies that did not refer to the molecular, genetic or cellular factors in peri-implantitis. Ultimately, 55 articles were included in the study for full-text reading. The full-text reading did not exclude any articles (Figure 1). The data from the eligible articles were extracted and handled by the two reviewers (MC and AP). A spreadsheet was created in Microsoft Excel (Microsoft Corporation; Redmond, WA, USA) in order to collect data on the papers’ time and type of publication, journal and results (Table 1).

## 3. Literature Search Results

### 3.1. Types of Peri-Implant Disease and Criteria for Implant Health and Peri-Implantitis

Peri-implant disease has been divided into three separate categories by the consensus report of the 4th workgroup of the 2017 World Workshop on the classification of periodontal and peri-implant diseases and conditions (Table 2). These are, respectively: peri-implant health, peri-implant mucositis and peri-implantitis [75]. This division allows for clearer distinction and easier treatment planning, as peri-implantitis is not always coexistent with visible inflammation and can be easily mistaken for peri-implant mucositis. Implant success is recognized differently by different authors. Various definitions have been formulated ever since the first osseointegration cases were published by Branemark et al. Buser et al. [76] defined implant success as lack of mobility, no noticeable radiolucency around the implant, <2 mm of crestal bone loss in the first year of functioning and no inflammatory symptoms. Furthermore, the authors highlighted the importance of the feasibility of restorations. Implant success definition was then further redefined in 2008, at the International Congress of Oral Implantologists (ICOI) Pisa Consensus Conference, as no pain or tenderness upon function, the absence of mobility, <2 mm radiographic bone loss from the initial surgery and no presence of exudate [77]. To date, the most recent definition of peri-implantitis includes the presence of bleeding/suppuration on probing, increasing probing depth between examinations, and crestal bone loss not caused by the initial remodeling [78,79]. The 2017 World Workshop on the classification of periodontal and peri-implant diseases and conditions formulated the following definition of peri-implantitis: “peri-implantitis is a plaque-associated pathological condition occurring in tissues around dental implants, characterized by inflammation in the peri-implant mucosa and subsequent progressive loss of supporting bone” [6,80]. Additionally, there are criteria for diagnosing peri-implantitis without previous radiographic and clinical implant history, which consist of a lack of bleeding/suppuration on probing, PD ≥ 6mm and bone levels ≥ 3 mm apical of the most coronal portion of the intraosseous part of the implant [75,78,79,80,81,82,83].

### 3.2. Risk Factors Associated with Peri-Implantitis

**Figure 2:**
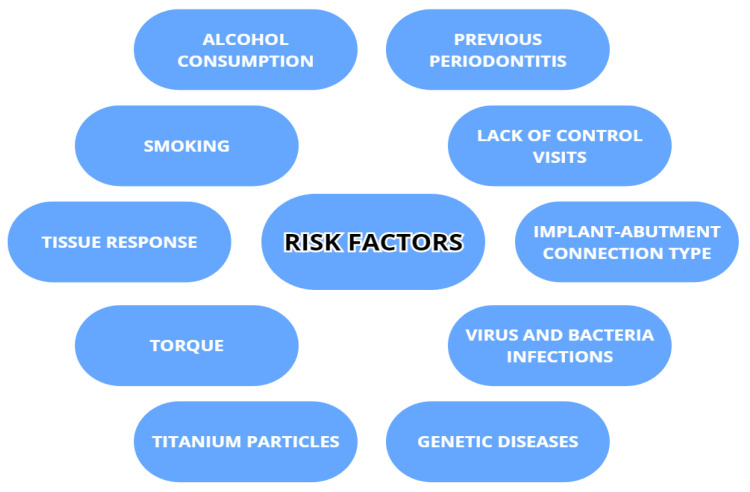
Risk factors associated with peri-implantitis.

The risk factor of peri-implantitis is dependent not only on individual host susceptibility, but also on other factors with various degrees of concurrence. To what extent the risk factors will influence the appearance of peri-implantitis also depends on the frequency, the intensity, individual vulnerability to the factor (i.e., a thicker peri-implant phenotype performs better) [22] and the cooperation of factors acting together. The risk factors include smoking, alcohol drinking, metabolic diseases (e.g., diabetes), previously recognized periodontitis [23,24,25,26,27,28], a poor level of oral hygiene, insufficiently frequent controls, an external implant–abutment connection type and inadequate screw-in torque [23], viral infections (HPV, HHV-4, HHV-6, HHV-7 and COVID-19) [28,29,30], genetic burdens (i.e., Papillon–Lefevre syndrome), titanium particles present after implant placement, and tissue response to prosthetic restoration [31,32]. Smoking is positively correlated with peri-implant disease as it can contribute to hindering the bone blood supply and lower the cellular immunological response and MMP-8 [22,24,26,32,33]. Alcohol drinking can also increase the risk of peri-implantitis, mainly in conjunction with smoking, highlighting the additive influence of both [22,24]. There is also a statistically significant risk of peri-implantitis in obese patients because of higher C-reactive protein and MMP-8 levels in the serum and PICF [32,33]. Patient compliance also plays an important role in the quick detection and effective management of implant tissues [24]. Patients with genetic conditions that can influence periodontal health, such as Papillon–Lefevre syndrome, are inherently more prone to peri-implantitis. In this group, despite the much higher risk, regular clinical controls either lowered or prevented peri-implantitis and its progress [34]. Regarding endocrine malfunctions, diabetes mellitus leads the way. Its growing significance comes from an ever-growing population of patients and new dependencies found in metabolic pathways and genetic connections. In peri-implantitis accompanying diabetes, the main reasons seem to be increased HbA1c and advanced glycemic end product (AGE) levels, which interfere with immune response, bone remodeling (promoting osteoclastogenesis), vascularization, cell apoptosis and inflammation [22,25,32,35]. Risk factors are listed in the Figure 2.

### 3.3. Molecular Factors Contributing to Peri-Implantitis Development

**Figure 3:**
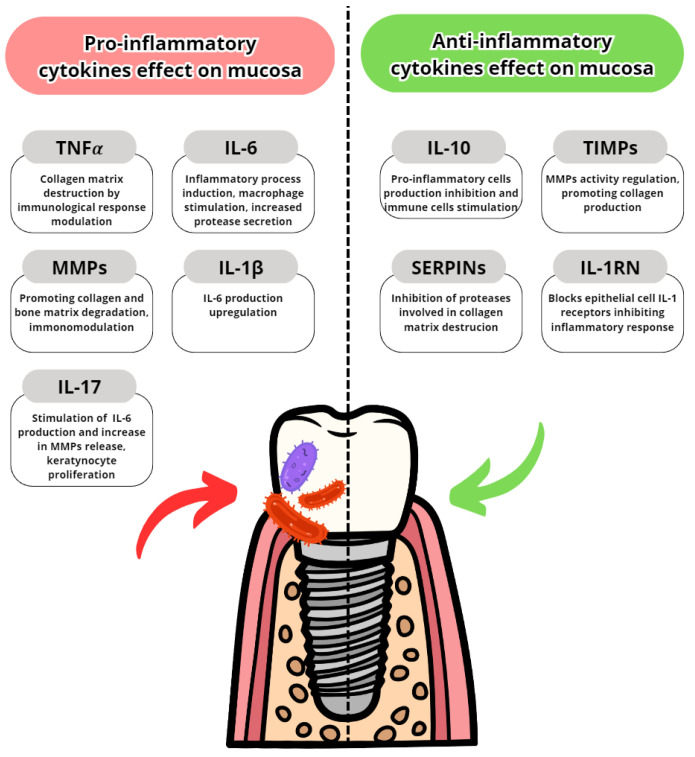
Pro- and anti-inflammatory cytokine effects on oral mucosa and gingiva [84,85,86,87,88,89,90,91].

**Figure 4:**
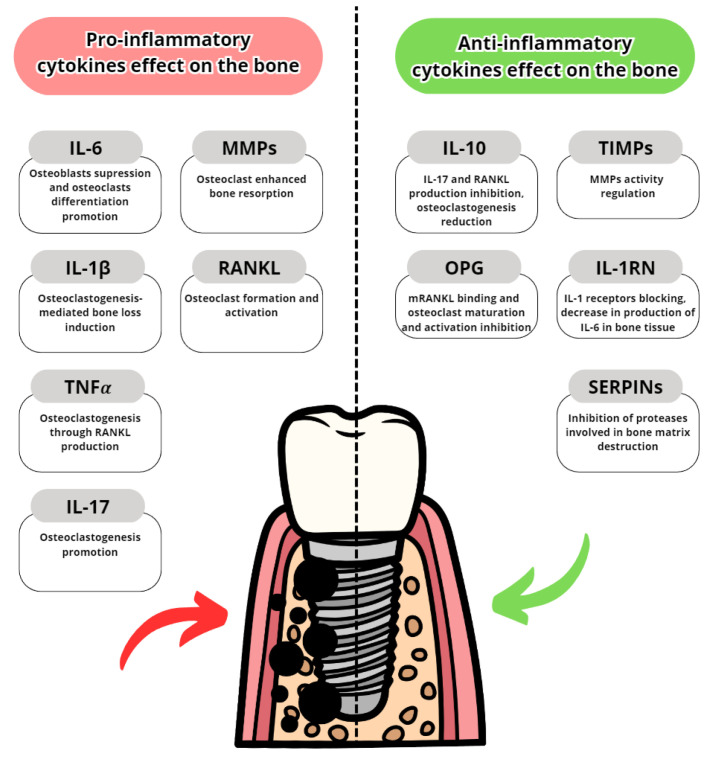
Pro- and anti-inflammatory cytokine effects on alveolar bone [84,85,86,87,88,89,90,91].

Cytokines are proteins secreted by leukocytes and serve a mainly communicatory role. They influence either pro- or anti-inflammatory responses. In peri-implantitis, the balance between pro-and anti-inflammatory cytokines is disrupted in favor of pro-inflammatory. The most well-known are pro-inflammatory IL-6, IL-1 and TNFα [7]. The most common pro- and anti-inflammatory cytokines are listed in Table 3 with their effects in peri-implantits in Figure 3 and Figure 4. The basic functions of the most common cytokines in peri-implantitis are listed in Table 4.

#### 3.3.1. Pro-Inflammatory Cytokines

##### Interleukin-6 (IL-6)

Interleukin-6 has been long known to be one of the core inflammatory cytokines. IL-6 can be excreted by osteocytes and can promote osteoclast formation. It also shows some anti-inflammatory mechanisms, hindering TNF-alpha effects. In peri-implantitis, the major sources of IL-6 are macrophages, which secrete IL-6 in response to specific microbial molecules through pathogen-associated molecular patterns (PAMPs) and serve a pro-inflammatory purpose. Its elevated levels are recognized in most inflamed tissues. In peri-implantitis, however, the concentrations of IL-6 in peri-implant cervical fluid (PICF) are more substantial. Subsequent research confirms this trend, with some studies extending the comparisons to peri-implant mucositis [25,35,36,37,38,39,40,41,42,43]. Among all the research found, only one study did not find distinctively elevated levels of IL-6. As surprising as the result was, it was highlighted that the research was conducted on a relatively small patient group [30]. Otherwise, increasing IL-6 levels are regarded as one of the main factors for the progression of peri-implantitis, and are difficult to keep under control with treatment. The differences between peri-implantitis and periodontitis regarding PICF, and salivary levels were minor and statistically insignificant [42]. However, when comparing peri-implant mucositis and peri-implantitis, there were significantly higher levels in the peri-implantitis group [37].

##### Interleukin-1β (IL-1β)

IL-1β is the main pyrogen cytokine. For activation, it needs caspase-1, as the IL-1β secreted by macrophages is initially inactive. The effects of IL-1β, which triggers other pro-inflammatory cytokines, position it as one of the core cytokines in the inflammatory response. Levels of interleukin-1, mainly beta, saw an increase in PICF in peri-implantitis [30,32,35,36,37,43,44,45,46,47,48,49]. There was a positive correlation of PD, PI, GI and BL with IL-1β levels, similarly to periodontitis [47]. What is also noteworthy is that the levels of IL-1β positively correlate with implant failures [32]. This makes IL-1β particularly interesting in the case of diagnostics, as it may serve as a predictor of the severity of peri-implantitis [25,32,36]. The research conducted by Fernandes et al. elevates IL-1β as the main factor in bone loss during peri-implantitis [36]. Research conducted by Sahoo et al. compared the levels of IL-1β between healthy and peri-implant patients and found a 3x increase in the test group [30]. IL-1β also shows more intensified secretion in the early stages of peri-implantitis, triggering catabolic changes in a very early stage [44]. Unlike IL-6, IL-1β does not present similar levels in peri-implantitis and in periodontitis. Moreover, its levels are lower than in mild periodontitis in some cases [45,49]. Additionally, contrary to IL-6, IL-1β levels are lower in peri-implantitis than in peri-implant mucositis, highlighting other possible factors contributing to bone resorptions [37].

##### Tumor Necrosis Factor α (TNFα)

TNFα is the third main inflammatory cytokine one can detect in inflamed tissues. Peri-implant tissues are characterized by overall elevated levels of TNFα, both in soft and hard tissues, compared to healthy implants [30,36,41,43,46,50]. Upon comparing the concentrations of TNFα between periodontitis and peri-implantitis, greater concentrations were noticed in the peri-implantitis group [40]. The main cells responsible for its secretion are epithelium cells, dendritic cells, fibroblasts, macrophages and neutrophils [37]. The amount of TNFα corresponds with the degree of bone destruction as it can influence osteoclastogenesis through RANKL. For this reason, as with IL-1β, it can serve a prognostic role in peri-implantitis progress [32]. However, research by Aleksandrowicz et al. noted, that in PICF, TNFα levels were the highest in medium peri-implantitis, with levels exceeding those in early periodontitis. However, they still put emphasis on the promising prognostic and detection aspects of PICF TNFα levels [45]. Elevated levels of TNFα were noted both in saliva and PICF, more often from PICF. For diagnostics, this could lead to an easier testing procedure using standard swabs for antigen tests, although more accurate measurements are collected from PICF. In terms of lowering the mean concentration of TNFα, the main factor is correct peri-implant maintenance therapy. A study by Gomes et al. noted that salivary levels of TNFα without proper maintenance therapy were higher and corresponded to exacerbated peri-implantitis in 5 years [51].

##### Interleukin-8 (IL-8)

IL-8 is a chemokine with one of the strongest neutrophil chemotactic abilities among these substances, and it promotes phagocytosis and degranulation. It is produced by neutrophils, macrophages, lymphocytes, basophils and epithelial cells. An increase in mean IL-8 tissue concentrations was noted, which was more substantial than in periodontitis groups [40]. As one of the main chemotactic agents, higher concentrations of IL-8 translate to more acute inflammatory infiltrate, thus allowing for greater bone resorption, as seen in peri-implantitis. However, this effect can be mitigated with proper treatment and hygiene. Fernandes et al. highlighted that the coronal inflammation modulated by peri-implant bacteria, more specifically, LPS, and the secretion of IL-8 are dependent on and regulated by matrix fibroblasts. If there are constantly high level of LPS, fibroblasts produce IL-1, -6 and -8, which then indirectly activate RANKL and osteoclastogenesis. On the other hand, reducing LPS activates anti-inflammatory mechanisms in matrix fibroblasts, which promote reconstruction [36]. Despite levels of IL-8 frequently being elevated, it is not always the case. Some studies indicate similar levels of IL-8 between healthy and peri-implant implants [43]. Interestingly, in patients with herpesvirus, the control group (no peri-implantitis) had more IL-8 detected in the PISF than the test group (with peri-implantitis) [30]. Unfortunately, the reason for this behavior is yet to be discovered.

##### Interleukin-17 (IL-17)

IL-17 is a pro-inflammatory cytokine with mainly signaling purposes. Its production by T-lymphocytes is stimulated by IL-23. The levels of IL-17 are elevated in peri-implantitis [36,37]. There have been assumptions as to whether IL-17 and TNFα levels are correlated, but Darabi et al. found no such link. IL-17 is also not correlated with sex or age. A positive correlation was found between probing depth and IL-17 [50]. The higher expression of IL-23 and lower levels of TGF-beta translated to a more acute response from Th17 lymphocytes [32,33]. However, upon comparing the levels of IL-17 in mucositis and peri-implantitis, Farhad et al. found the highest mean concentrations of IL-17 in the mucositis group. Both the mucositis and peri-implantitis groups’ levels were significantly elevated compared to those of healthy patients. Research conducted by Gao et al. considered ethnicity. In their research, there were Uygur and Han patients. In the healthy group, there were no significant differences in IL-17 between the two, whereas in peri-implantitis, the IL-17 levels in the Uygur test group were significantly higher than in the Han test group [48]. These results highlight the differences in difficulty in managing the Th17-cell response depending on the ethnic origin.

##### Collagenase-2 (MMP-8/aMMP-8)

Collagenase-2 (MMP-8), or neutrophil collagenase (highlighting the origin), is an enzyme that targets mainly collagen type I and III. Its enzymatic properties are dependent on zinc and calcium ions. MMP-8 is the most prevalent metalloproteinase found in both GCF and PICF. It is often considered a major mediator in aggressive tissue destruction, and often correlates positively with disease severity and osteolysis [37]. Peri-implantitis is no exception and has elevated levels of MMP-8 and the active form-aMMP-8 [1,32,34,36,37], which are more intensified in peri-implantitis than periodontitis [40,46]. Only one study did not find a noticeable difference between periodontal and peri-implant tissues [44]. aMMP-8 also positively correlates with subgingival bacterial colonization (mainly *P. gingivalis*) [33]. Furthermore, aMMP-8 is relatively easy to monitor, so it can be used as a diagnostic risk factor with good sensitivity. In comparison, non-activated MMP-8 has smaller diagnostic potential than its activated form. In one study, a quantitative aMMP-8-PoC test achieved 78% accuracy in a non-adjusted model and 80.6% in an adjusted quantitative aMMP-8-PoC test. Only calprotectin showed better specificity, but only in a non-adjusted model [39]. In another study, the results of an immunofluorometric assay (IFMA) PoC/chairside test were even better, showing 76–90% sensitivity and up to 96% specificity [52]. A commonly adopted scale for assessing an aMMP-8-PoC test is: <20 ng/mL minimal risk, 20–80 ng/mL elevated risk and >80 ng/mL high risk [39]. Another study compared ELISA and an Immuno-Assay test for aMMP-8, and the authors found that the Immuno-Assay performed better, and the diagnostic importance of aMMP-8 was strongly highlighted, no matter what diagnostic technique the doctor used [33]. Although aMMP-8 alone has a strong diagnostic position, combining it with TIMP (TIMP/MMP-8 ratio) and ICTP (MMP-8/ICTP) presents even more accurate results. This combination is also less susceptible to diagnostic error due to patient smoking, as aMMP-8 and MMP-8 levels show a decrease in long-term smokers [33].

##### Other Metalloproteinases and Activated Metalloproteinases (MMPs/aMMPs)

There are other metalloproteinases present in peri-implantitis, although they are not always present in elevated levels. Research by Lähteenmaki et al. found that MMP-2 and MMP-9 levels were often indistinguishable between healthy implants and peri-implantitis [39]. Such results were also achieved by Figueiredo et al [42]. On the other hand, research by Baseri et al. found elevated levels of MMP-2 and -9 [31]. MMP-9 is especially tricky to assign a certain role in peri-implantitis, because when the levels are found to be high, together with MMP-8 and -13, there tends to be more bone loss around the diseased implant [37]. Therefore, whether MMP-9 and -13 can have a modulatory effect on MMP-8 and enhance its effects on bone resorption needs to be further verified. When it comes to MMP-7, Baseri [31] and Fernandes et al. [36] also indicated an increase in MMP-7, but Correa et al. found a decrease [32]. Interestingly, levels of MMP-2 in a group without regular peri-implant preventive maintenance were higher in peri-implantitis than healthy implants, whereas in a group with regular peri-implant maintenance, MMP-2 levels were lower and similar between healthy implants and those affected by peri-implantitis [51]. This shows the need for regular peri-implant maintenance therapy in terms of prevention and treatment. MMP-13 is rarely mentioned in the literature screened for this study. In an article comparing Uygur and Han populations, MMP-13 was significantly higher in the Uygur control group than in the Han group, indicating ethnic differences in cytokine expression [48].

#### 3.3.2. Anti-Inflammatory Cytokines

##### Interleukin-10 (IL-10)

Interleukin-10 is an anti-inflammatory cytokine. It is mainly produced by monocytes as a response to cervical bacterial flora. An adequate response from monocytes can influence the expression of Th1 and both prolong and enhance tissue proliferation. Peri-implantitis is a somewhat interesting case of the delicate balance of cytokines in this disease. Some authors describe elevated levels of IL-10, even with elevated levels of IL-1 and TNFα at the same time [32,43]. Others indicate a noticeable decrease in IL-10 concentrations [40], or an unchanged concentration but a disturbed IL-1β/IL-10 ratio, favoring a pro-inflammatory process [44]. Interestingly, when comparing peri-implantitis with peri-implant mucositis, the former presented higher concentrations of IL-10 in tissues surrounding implants [49,53]. Upon comparing this with the elevated levels of IL-17 measured in the same study, peri-implant mucositis emerged as the more acute form of inflammation [52]. In order to find a definitive answer to why both pro- and anti-inflammatory cytokine levels are elevated, there would need to be more studies regarding soft and hard tissue activity, blood supply and cellular sensitization to cytokines. Currently, more articles highlight the decrease in IL-10 levels in PICF, with a negative correlation with diseased implants [37].

##### Tissue Metalloproteinase Inhibitors (TIMPs)

Tissue inhibitors of metalloproteinases (TIMPs) are specific inhibitors designed to restrain the effects of metalloproteinases on tissues. In peri-implantitis, the general trend is the downregulation of TIMP synthesis. Main TIMP found in peri-implant tissues is TIMP-1, whose levels are mainly noted as decreased. It hinders the activity of MMP-1, MMP-7 and MMP-8 [32], slowing down the destruction of collagen type I. Even if the differences in levels of MMPs are small compared to healthy implants [42], the MMP/TIMP-1 ratios are often unbalanced, favoring destructive mechanisms [40]. As MMP-1 and MMP-8 are mainly responsible for collagen matrix destruction in peri-implantitis, the MMP-1/TIMP-1 and MMP-8/TIMP-1 ratio shifts in favor of MMPs can serve as an indicator of peri-implantitis progression and inefficiency of treatment [37]. Another TIMP found in peri-implant tissues was TIMP-2, which is also downregulated in peri-implantitis [40], but it had a less important role in peri-implant disease than TIMP-1.

##### RANKL, OPG and RANKL/OPG Ratio

The Receptor Activator of Nuclear Factor kappa-B ligand (RANKL) and osteoprotegerin (OPG) are essential molecules in bone turnover and homeostasis. RANKL is responsible for osteoclastogenesis, whereas OPG inhibits osteoclasts maturation. Osteoclastogenesis that occurs through RANKL is both a direct and indirect type of bone loss mechanism and is dependent both on RANKL excretion and the osteoclast response [36]. An increase in RANKL with a decrease in OPG is the most seen scheme in peri-implantitis [32]. The balance of RANKL/OPG in peri-implantitis is influenced by TNFα and NFkB in favor of RANKL [31,54]. There is also a TLR4 signaling pathway that may influence B-cell infiltration and the RANKL/OPG ratio in peri-implantitis, thus modulating the inflammatory response [55]. A rise in soluble RANKL (sRANKL) in saliva can be relatively easily detected with commercially available tests and can exacerbate osteoclastogenesis [40]. It must be noted that sRANKL levels are not always elevated in peri-implantitis, and a comparison with OPG levels is needed to determine whether RANKL/OPG balance is disrupted [36]. Proper implant treatment and hygiene can restore the balance between RANKL and OPG, thus reducing osteoclastogenesis and introducing homeostasis [51]. This allows for reparative processes to rebuild the damage to some extent. Unfortunately, the inflammation in deeper parts of the implant often requires surgical strategies, as stimulation with TNFα keeps the osteoclastogenesis process going.

### 3.4. Genetic Differences Increasing Risk of Peri-Implantitis

**Figure 5:**
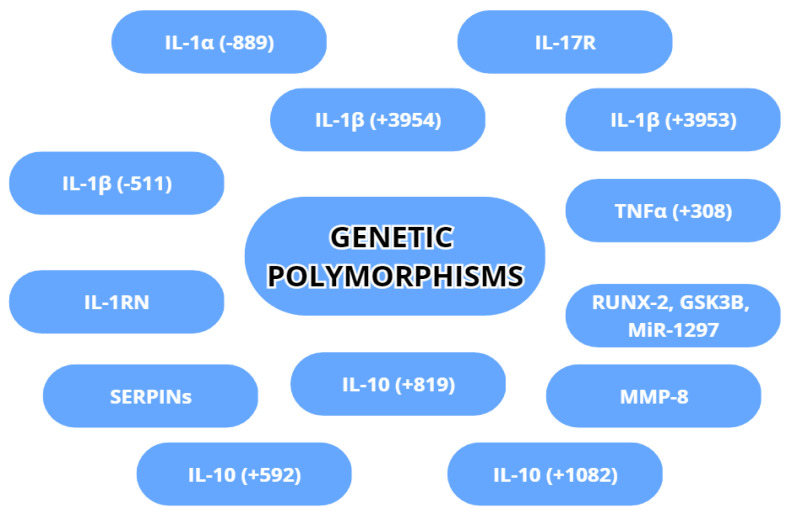
Genetic polymorphisms with possible association with peri-implantitis.

Genetic variability is an inherent factor in any species and can affect even the best-developed treatment for a disease. In peri-implantitis, some gene variations have implications for the susceptibility or acceleration of destruction in peri-implant tissues. The most commonly examined gene variations are genetic polymorphisms regarding IL-1α, IL-1β, TNA-α, MMP-8 and IL-10. The genetic polymorphisms are shown in Figure 5.

#### 3.4.1. IL-1β (+3953/+3954)

The Interleukin-1B genetic polymorphism is well documented in the literature as it concerns one of the most important cytokines in peri-implantitis. It was found that there was a correlation between IL-1β +3953, together with IL-1α (−889), and an increased risk of peri-implantitis [56]. For the IL-1β +3953 polymorphism, however, further studies are needed on a broader patient group, as the current results are based on the selection of certain individuals with specific composite genotypes, rather than checking if genotype polymorphism increases susceptibility. IL-1β +3954 is also correlated with peri-implant disease, but the results of these studies are heavily dependent on sample sizes (number of samples and sample size) [56,57,58]. The +3954 variant is even becoming recognized as a negative treatment factor, since the inflammation is more intense, and the likelihood of implant loss is found to be increased [56]. An interesting element is the occurrence of mixed polymorphisms of IL-1α and IL-1β. Jin et al. found that deviation from the common genotypes of IL-1α and I-1B and their mixed polymorp hism increased the chance of peri-implantitis occurrence by 1.95×, and more intense bone loss around an implant by 1.76× [57]. Thus it can be concluded that IL-1α (−889) with IL-1β (+3954) is a negative predictor of both progression and treatment in PID [59,60]. This effect is further intensified by heavy smoking (>20 cigarettes a day) [59,60]. As the data collection and diagnostic methods develop, the incidence of polymorphisms and “risk allele” discovery increases. In some groups, it reaches up to 54% [23], and it is higher in PID group compared to healthy patients [61].

#### 3.4.2. IL-1β (−511)

Another interleukin-1β polymorphism that influences peri-implantitis is IL-1β (−511) [32]. Data correlating the increase in PID in this case are not as straightforward. The presence of IL-1β (−511) was found to be dependent not only on sample size, but also on ethnicity and PID duration [56]. The effects of IL-1β (−511) on peri-implant tissues are also more noticeable in heterozygotic patients and when there are the type TT and CC genotypes of IL-1β [57,60]. It remains to be investigated whether this polymorphism type influences PID incidence or is the result of PID.

#### 3.4.3. IL-1α (−889)

Interleukin-1A (−889) is the main IL-1α variation that corresponds to an increased risk of peri-implantitis. As mentioned above, its mixed polymorphism with IL-1β (+3953) or IL-1β (+3954) greatly increases the harmful potential of both cytokines [56,57,58,59,60]. Its diagnostic results are dependent both on genetic material sample size and ethnicity. Furthermore, the allelic variation within IL-1α (−889), such as the CT and TT types in the Asian population, can further influence tissue destruction [57]. The development of easier diagnostic procedures and protocols may allow for better and cheaper diagnostics, especially for mixed IL-1α/IL-1β genotypes, thus allowing for the “tailor made” treatment of peri-implantitis and the early screening and maintenance of high-risk patients.

#### 3.4.4. TNFα (+308)

The main TNFα polymorphism in peri-implantitis is TNFα (+308). The GA, AA and A/G genotypes of the TNFα (+308) polymorphism are more frequently found in peri-implantitis and are associated with an increased risk of implant failure [26,32,58,60]. It may seem that TNFα (+308) has an effect on PID, but this is not always the case. Other studies debate that TNFα (+308) does not always present an effect on peri-implantitis and needs other factors, such as smoking, to potentiate the TNFα (+308) genotype [57,62]. Another study showed that allele type GA presented an elevated risk of PID only in the Asian population [62], and other alleles did not present an elevated risk of PID. Unfortunately, these studies were not carried out on large patient groups and lacked standardized diagnostic methods, thus introducing a lot of bias in the obtained results.

#### 3.4.5. MMP-8

MMP-8 is an enzyme that will be discussed later in more detail. As one of the core enzymes in peri-implantitis, polymorphisms can greatly affect the development of PID. The genetic upregulation of MMP-8, detected via fluorescence-activated cell sorting analysis, influences other cytokines and enzymes [63] and promotes bone defect formation. Because the genetic pathway of MMP-8 in the end influences many more molecular factors, MMP-8 levels tend to stay at a higher level, even with proper and effective treatment [23]. Thus far, there is little information about specific polymorphisms of MMP-8 and peri-implantitis, as interest has shifted to the diagnostic ability of MMPs in inflammation. With time and further studies, high-risk genotypes shall be defined, as aMMP-8 is relatively easy to detect and collect from saliva and genotype differences can influence treatment protocols.

#### 3.4.6. IL-10 (+1082), (+819) and (+592)

Interleukin-10 genotypes are also associated with peri-implantitis, mainly with hindered IL-10 anti-inflammatory response. First and foremost is the IL-10 (+1082) genotype, which is considered to be associated with peri-implantitis development. Petkovic-Curcin et al. found that IL-10 (+1082) type GA/GG is discovered more often in patients with peri-implantitis [26]. This result is in contrast to Jamshidy et al.’s research, in which IL-10 (+1082) was not associated with PID and was not much different from the standard IL-10 form. IL-10 was also found to have (+819) and (+592) genotypes, but the results were insufficient to attribute them to increased risk factor in PID [62].

#### 3.4.7. IL-17R and IL-1RN

The interleukin-1 receptor antagonist (IL-1RN) gene regulates the synthesis of IL-1α and IL-1β receptor competitor IL-1RA. Controlling the receptor site and binding it allows for excess IL-1α and IL-1β to be discarded. The genotype polymorphism IL-1RN (VNTR), in recent studies, has shown no clear association with the increased risk of PID [56], although functional genetic polymorphisms of IL-1β (+3954) and IL-1RN (VNTR) may diversify IL-1β and IL-1RA protein production, thus affecting inflammation and causing implant failure [56,61]. This further highlights the negative effect that specific mixed genotypes have on peri-implant tissues. For IL-1RN, heavy smoking can also potentiate the IL-1RN (VNTR) genotype and greatly increase PID risk compared to smoking or IL-1RN (VNTR) alone [61].

The less-studied IL-17 receptor (IL-17R) expression was found to have no association with the development and risk increase of peri-implant disease [64].

#### 3.4.8. SERPINs

Serine Protease Inhibitors (SERPINs) are common inhibitors found in tissues. Unlike other protease inhibitors that competitively occupy protease active sites, SERPINs irreversibly inhibit the targeted protease, disturbing active sites. Bioinformatic analysis discovered four SERPINBs (B1, B3, B4 and B5) with significantly elevated expression in peri-implantitis compared to the control groups, and SERPINBs’ presence in PICF was noted. The lowest AUC detected in PICF was for SERPINB5, and the highest mean AUC was for SERPINB4. SERPINB1 and SERPINB3 were detected with significantly higher ROC-AUC, reaching between 0.8478~0.9565 and 0.8798~0.9738, respectively, with a confidence interval of 95%. This leads us to believe that SERPIN activation detection could have a promising role in the diagnosis of peri-implantitis. The four mentioned SERPINB expressions are also positively correlated with IL-6 and TNFα levels in inflamed tissues, with the highest correlation noted for SERPINB1. In this context, SERPINBs with cytokines may modulate the progression of peri-implant disease [41].

#### 3.4.9. Other Important Genetic Factors: RUNX-2, GSK3B and MiR-1297

There are many other genetic factors whose expressions in peri-implantitis have a different form of regulation [65]. One of these is the RUNX2 gene, which is responsible for the RUNX2 transcription protein associated with osteoblast differentiation. The RUNX2 gene in peri-implantitis is downregulated compared to healthy controls [32,66]. This change inhibits osteoblast formation in response to bone destruction. This has a profound effect on the bone repair process, which is often additionally interrupted by stimulated fibroblasts [32,63]. As a result, a peri-implantitis implant connection has a fibro-osteoblastic nature, restraining the osseointegration process and translating to a higher risk of implant loss [63]. In peri-implantitis diagnostics and RUNX2 downregulation, functional overload plays an important role in the treatment of peri-implant disease [36]. Overload correction brings RUNX2 expression to levels more like those of a healthy implant and, with proper treatment, limits unfavorable conditions of bone defect regeneration.

Furthermore, some RNA material seems to be linked to higher peri-implantitis incidence. The RNAs found to be responsible for the immune microenvironment were GSK3B and MiR-1297. They may play a significant role in the pathogenesis of peri-implantitis, although further genetic studies are needed [67].

#### 3.4.10. Peri-Implantitis’ Genetic Link to Other Diseases

With advances in genetic diagnostics, the expansion of databases and improved understanding of the links of peri-implantitis with other diseases, some common genes have been discovered. Three of these—IL-6, NFkB and PIK3CG—have been linked with peri-implantitis and diabetes mellitus type 2 [68]. There have also been links found between peri-implantitis and Alzheimer’s disease, as two DEGs have been found to be unregulated in both conditions [54]. This may lead to the genetic upregulation and increased susceptibility of peri-implant patients to either type 2 diabetes or Alzheimer’s disease.

### 3.5. Cellular Factors

The main cells present in the vicinity of implants are fibroblasts, neutrophils, osteocytes, macrophages and epithelial cells. Peri-implantitis is characterized by increased connective and pocket epithelial tissues levels [69]. Inflamed connective tissue shows high disorganization with great inflammatory cell infiltration (mainly plasma cells and neutrophils). Collagen matrix fibers often indicate a lack of accompanying fibroblasts, which show modulatory effects on inflammation processes and the reconstruction of collagen [36]. Instead, there is inflammatory destruction of the collagen skeleton. The prevalence of collagen types has shifted from collagen type I to collagen type III [66]. Changes noticed on microscopic cross-sections were greater than in periodontitis. An analysis of grafted peri-implantitis connective tissues showed the expression of CD68, MPO and iNOS surface proteins. On the other hand, 8-deoxyguanosine (8-OHdG) markers, both nuclear and mitochondrial, showed a decrease in peri-implantitis compared to periodontitis, indicating decreased heat-shock RNA destruction. In peri-implantitis epithelial cells, membranous proteins expression showed differences in the levels of γ-H2AX, iNOS, NOX2, MPO and the PAD4/MPO ratio [69]. Interestingly, not every study detected changes between the control group and peri-implantitis in the peri-implant epithelium [75]. As for immunological cells, the area of inflammation in peri-implantitis consists of commonly found neutrophils, plasma cells, macrophages and T-cells. Neutrophils particularly densely infiltrate the coronal part of the implant, whereas deeper parts show a higher number of M1-type macrophages and a shift in the M1/M2 ratio [31,70,71]. The shift in the balance of M1/M2 macrophages can play a role in the osteolytic effect and progression. It has also been noted that while exposed to PDLF, macrophages reduced the excretion of TNFα significantly and of IL-1β minimally [72]. This behavior of macrophages shows interesting directions in which we can modulate an inflammatory response [37]. A coexisting rise in the number of dendritic cells can also negatively affect Langerhans cell maturation and modulation of the intensity of inflammation [31]. The most important cells in peri-implantitis are listed with their function in Table 5.

### 3.6. Diagnostic Opportunities (aMMP-8, TNFα, IL-1β, IL-6)

The spectrum of diagnostic molecules in peri-implantitis that is taken into consideration has broadened in recent years. MMP-8 clearly leads the way in peri-implantitis screening and even grading. One of the most often used tests in research is the point-of-care/chairside test. The measurements for peri-implantitis of aMMP-8 are: <20 ng/mL = low risk, 20–80 ng/mL = elevated risk and >80 ng/mL = high risk. The screening of MMP-8 can help in the early stages of peri-implant mucositis and peri-implantitis, allowing for quick therapeutic intervention [73]. The level of MMP-8 decreases with correct treatment and can be used for treatment evaluation [23]. While using the point-of-care/chairside enzyme test, aMMP-8 has the highest sensitivity when compared with IL-6 [39,53,74]. However, it must be said that for MMP-8 (and IL-1β and IL-6, too), the PoC/chairside test is dependent on the PD of the diseased implant. Other studies compared ELISA and an Immunoassay for MMP-8 screening, with the Immunoassay performing better than ELISA [33]. MMP-8 is also a promising biomarker for peri-implant osteolysis around diseased implants [37]. Another important biomarker in diagnostics is IL-1β. IL-1β levels in peri-implantitis are elevated from the very beginning; hence, it can be used for diagnostic purposes before clinical manifestations [31,46,55,92,93]. It is detected in the same way as MMP-8, that is, using the PoC/chairside test [33]. Moreover, IL-1β levels positively correlate with lost implants [32]. Periodic clinical controls facilitate the detection of a transition to more severe peri-implant disease stages [44]. TNFα has similar diagnostic properties to IL-1β [32,55], but shows less specificity. TNFα is also tricky to classify, due to the difficulty in setting benchmark levels, as smaller TNFα concentrations are recognized in peri-implantitis than medium-grade periodontitis [45]. Last, but not least, is IL-6. Thus far, it is the least used cytokine of those mentioned above in peri-implantitis diagnosis. It can be detected using the PoC/chairside test, but the results have higher diagnostic bias compared to MMP-8 [39]. Nonetheless, it may provide sufficient data for peri-implant recognition, especially with a deep probing depth noted [46].

## 4. Conclusions

There has been a lot of new research conducted in the field of the molecular, genetic and cellular factors in peri-implantitis. The evidence suggests that currently, the most important pro-inflammatory cytokines are Il-6, IL-1, TNFα and MMP-8. Nevertheless, other cytokines can enhance the effects of those mentioned here. They potentiate the cellular response and are commonly extracted from PICF. The genetic variability of cytokines also makes patients more susceptible to peri-implantitis incidence and makes the treatment outcome unfavorable. Combining these factors with cellular imbalance caused by general peri-implantitis factors, such as smoking, previous periodontal conditions, diabetes mellitus, improper restorations and a lack of proper maintenance, makes the development of peri-implantitis a rapid process resulting in implant loss. However, cytokines can also act as a diagnostic tool. Currently the most accurate and sensitive diagnostic cytokine is MMP-8/aMMP-8, extracted from PICF. Furthermore, genetic polymorphisms and different cytokine expressions are promising paths for novel drugs to act on, though more research on this matter is still needed.

## Figures and Tables

**Table 1:** Literature search results.

Number [Citation]	Authors	Journal	Date of Publication	Type	Results
1 [7]	Liu C. et al.	Advanced Science	2021	Review	Most well-known pro-inflammatory cytokines are IL-1, IL-6, IL-8, IL-12, IL-18, IFN-α/γ, and TNF- α. The anti-inflammatory cytokines are IL-1 RA, IL-4, IL-6, IL-10, IL-11, IL-13, and TGF-β. There are many ways of cytokine detection like ELISA assay, PoC testing, multiplex detection, flow cytometry, Luminex assays and MSD assays.
2 [22]	E Silva R. et al.	Brazilian Dental Journal	2020	Article	Smoking, alcoholism, lack of hygene, type 2 diabetes and thin gingival biotype increase the risk of peri-implantitis. RANK, RANKL and OPG polymorphisms did not increase the risk of peri-implantitis in amazon population.
3 [23]	Thierbach R. et al.	Journal of Clinical and Diagnostic Research	2016	Article	Although treatment decreases MMP-8 levels, the inflammatory levels are still present. Risk alleles were present in 54% examined patients.
4 [24]	Astolfi V. et al.	International Journal of Environmental Research and Public Health	2022	Article	Smoking, lack of regular follow-up visits and previous periodontal problems increase the chance of peri-implantitis, external abutment connection increases the risk of peri-implantitis
5 [25]	Plemmenos G. et al.	Life	2022	Review	Hyperglycemia and smoking have an adverse effect on peri-implant tissues, type 2 diabetes promotes AGE production, past periodontal problems make peri-implant tissues more sensitive to AGE and oxidative stress
6 [26]	Petkovic-Curcin A. et al.	The International Jounral of Oral & Maxillofacial Implants	2017	Article	Smoking and previous periodontitis increase the chance of peri-implantitis. Patients with peri-implantitis had more frequently IL-10 (−1082), IL-1RN and TNFα (308) genetic polymorphisms. Smoking elevates the risk if combined with mentioned polymorphisms.
7 [27]	Insua A. et al.	Journal of Biomedical Materials Research	2017	Narrative Review	The effects of systemic levels of cholesterol, fatty acids and vitamin D may be responsible factors for early implant loss and long-term implant stability. Immune cells have prolific impact on dental implant osseointegration and maintenance.
8 [28]	D’Ambrosio F. et al.	Dentistry Journal	2022	Review	Tobacco smoking, alcohol consumption, unhealthy diets, chronic stress and depression are promoting dysbiosis, immunologic deficiencies and inflammatory environment, increasing the chance of peri-implantitis. SARS-CoV-2 virus promoted disregulated production of especially IL-1beta, IL-6 and IFN-γ.
9 [29]	Mancini L. et al.	Frontiers in Oral Health	2022	Mini Review	COVID-19 infection may increase the production of MMP-8, leading to increased chance of peri-implant diseases when combined with poor oral hygene
10 [30]	Sahoo S.K. et al.	Journal of Pharmacy & Bioallied Sciences	2021	Article	In peri-implantitis IL-1β level is greatly increased. IL-8 and MMP-1 is decreased compared with healthy patients. Herpesviridae (HHV-4, HHV-6 and HHV-7) increase the expression of pro-inflammatory cytokines.
11 [31]	Baseri M. et al.	BioMed Research International	2020	Review	Increase in local macrophage and T-limphocytes may be due to type 4 hypersensitivity towards titanium particles, in periimplantitis macrophages balance shift towards pro-inflammatory M1 form, levels of IL-1β, IL-2, IL-8, IL-17, MMP-7, MMP-8, MMP-9 are increased in peri-implantitis, the RANKL/OPG ratio favours osteoclastogenesis, IL-1β can be used as earl diagnostic cytokine
12 [32]	Corrêa M. et al.	Brazilian Oral Research	2019	Review	In peri-implantitis there are elevated levels of IL-1β, TNFα, IL-8, IL-17, IL-23, MMP-1, MMP-8, RANKL and decreased levels of PPARγ, IL-10, TIMP-1, OPG, BMP-7, RUNX, IL-1RA. Furthermore, the polymorphisms of IL-1β (−511), MMP-1 (G-1607GG), TNFα (308) may increase the risk of peri-implantitis. TNFα and IL-1β can have diagnostic potential.
13 [33]	Al-Majid A. et al.	International Journal of Dentistry	2018	Review	aMMP-8 can detect subclinical periimplantitis stages, MMP-8 levels correlate positively with the bacteria infection, TIMP/MMP-8 tests are more sensitive and precise than sole MMP-8 tests, smokers have decreased levels of MMP-8, obesity increases MMP-8 levels
14 [34]	Nickles K. et al.	Journal of Clinical Medicine	2022	Case Report	In patients with Papillon-Lefevre syndrome, regular dental appointments delay the peri-implantitis
15 [35]	Al.-Askar M. et al.	Medical Principles and Practise	2018	Article	Increased Hb1c levels and improper oral hygene lead to increased IL-1β and IL-6 levels in saliva.
16 [36]	Fernandes M.H. et al.	Journal of Oral and Maxillofacial Research	2016	Review	Bone loss process is both direct and indirect. IL-1β, IL-6, TNFα, IL-17, IL-12, IL-8 are elevated, whereas IL-4, OPG and IL-10 are decreased. RUNX2, BMP-7 production is decreased, MMP-8 and MMP-7 are increased. Fibroblasts can restrain the immunological response, however LPS increases the production of IL-1, IL-6 and IL-8.
17 [37]	Alassy H. et al.	Diagnostics	2019	Review	PICF in peri-implantitis contains more aMMP-8, IL-6, TNFα and IL-1β. IL-10 levels decreased in peri-implantitis. The disproportion between MMP-1/TIMP-1 and MMP-8 levels can serve for diagnostic purposes. IL-1β levels can serve as predictor marker for peri-implantitis. Peri-implantitis tissues contain more CD138, CD68 and MPO-positive cells than in periodontitis.
18 [38]	Martins L. et al.	International Journal of Environmental Research and Public Health	2022	Brief Report	Increased level of AhR, IL-6 and GAPDH gene expression in peri-implantitis group
19 [39]	Lähteenmäki H. et al.	Clinical and Experimental Dental Research	2022	Article	The levels of IL-6, MMP-8, aMMP-8, calprotectin was elevated in peri-implantitis, MMP-8 has the best diagnostic value from the measured cytokines
20 [40]	Kalsi A.S. et al.	Journal of Periodontal and Implant Science	2021	Review	In peri-implantitis there are elevated levels of RANKL/sRANKL, TNFα, IL-6, IL-8, MMP-8, MMP-1 and decrease in IL-10, TIMP-1, TIMP-2, OPG, IL-10. Additionally, aquaporin-1 disregulation inhibits cell growth and cell homeostasis.
21 [41]	Jiang J. et al.	Journal of Clinical Laboratory Analysis	2021	Article	SERPINs taking part in inflammatory process in peri-implantitis are SERPINB1, SERPINB3, SERPINB4 and SERPINB5. The increase in IL-6 and TNFα is positively correlated with SERPINs level increase.
22 [41]	Jiang J. et al.	Journal of Clinical Laboratory Analysis	2021	Article	SERPINs taking part in inflammatory process in peri-implantitis are SERPINB1, SERPINB3, SERPINB4 and SERPINB5. The increase in IL-6 and TNFα is positively correlated with SERPINs level increase.
23 [42]	Figueiredo L. et al.	International Journal of Environmental Research and Public Health	2020	Article	IL-1β levels are significantly more elevated in peri-implantitis than in periodontitis or control group. IL-6, TNFα, MMP-1, MMP-8, MMP-2, MMP-9, TIMP-1 and TIMP-2 levels are similar between peri-implantitis and periodontitis.
24 [43]	Ata-Ali J. et al.	BMC Oral Health	2015	Article	Peri-implantitis group showed increased levels of IL-1β, IL-6, TNFα. The IL-1β/IL-10 ratio was significantly higher in peri-implantitis. Improper microbial control can lead to increased bone loss through inflammation.
25 [44]	Gleiznys D. et al.	Medical Science Monitor	2019	Article	In peri-implant tissues IL-10 levels decrease and IL-1β increase. Highest increase of IL-1 is in the beginning of inflammatory process.
26 [45]	Aleksandrowicz P. et al.	BMC Oral Health	2021	Article	The levels of IL-1β and TNFα are higher in periodontitis than in peri-implantitis, CXCL8 is dependent on the stage of periodontitis/ peri-implantitis and on patient susceptibility. Both IL-1β and TNFα can be used as diagnostic markers in periodontics
27 [46]	Ghaasib I. et al.	Clinical Implant Dentistry and Related Research	2019	Systematic Review	Il-1β, IL-6, TNFα and MMP-8 levels were elevated in PICF. IL-1β and IL-6 level measurements can be used to differentiate healthy patients, mucositis and peri-implantitis.
28 [47]	Yaghobee S. et al.	Journal of Dentistry of Teheran University of Medical Science	2013	Article	IL-1β levels positively correlate with PD, GI, BL, PL. The levels of IL-1β in PICF is higher than in GCF.
29 [48]	Gao X. et al.	Medicine	2018	Observational Study	Higher levels of IL-1β and MMP-8 were detected in Han population than in Uygr population, whereas MMP-13 levels was higher in Uygr population. This may lead to different progression of peri-implantitis between the two groups.
30 [49]	Eckert M. et al.	Molecular Oral Microbiology	2018	Pilot Study	Comparing teeth with implants, the levels of IL-1β in periodontitis exceeded those in peri-implantitis. The anti-inflammatory IL-10 was lower in periodontitis and peri-implantitis in comparison with gingivitis and mucositis. Expression of miropsin-1 was positively associated with levels of IL-1β and negatively associated with those of IL-10. Additionally, miropin may play a regulatory role in a multispecies dysbiotic biofilm forming on teeth and implant surfaces and may contribute to the initiation and/or progression of both periodontal and peri-implant diseases.
31 [50]	Darabi E. et al.	Iranian Journal of Allergy, Asthma and Immunology	2013	Article	TNFα and IL-17 levels were greatly increased compared with healthy patients, IL-17 levels are positively correlated with probing depth
32 [51]	Gomes A.M. et al.	Journal of Applied Oral Science	2019	Article	The peri-implant maintenance therapy significantly lowered TNFα levels. Increased salivary TNFα levels was associated with worse peri-implant clinical condition.
33 [52]	Alassiri S. et al.	Disease Markers	2018	Review	Periimplantitis site has elevated MMP-8 levels, MMP-8 PoC/Chairside tests have 76–90% sensitivity and 96% specificity
34 [53]	Farhad S.Z. et al.	International Journal of Preventive Denstistry	2019	Article	The highest levels of IL-17 were detected in peri-implant mucositis, followed by peri-implantitis. IL-10 level was the highest in peri-implantitis group.
35 [54]	Zhang H. et al.	Experimental and Therapeutic Medicine	2017	Article	IL-6, TLR-4, N1, IL1β, MMP9, CXCL8, CXCR4, CXCL1, PECAM1, and SPP1 genes are upregulated in peri-implantitis. Key genes in peri-implantitis are IL-6 and IL-1β
36 [55]	Zhang X. et al.	BioMed Research International	2021	Research Article	In periimplantitis there are 2 upregulated DEGs that are also upregulated in Alzheimer's Disease, HSP90AA1 and NFκB are also upregulated modulating osteoclasts, OPG and RANK
37 [56]	Mohammadi H. et al.	Pathogens	2021	Review	Periimplantitis is noted more frequently if IL-1β (+3954), IL-1α (−889) are present.
38 [57]	Jin Q. et al.	PLoS ONE	2021	Research Article	IL-1α (−889), IL-1β (+3954) and IL-1β (−511) may be more frequently found in periimplantitis. TNFα genotypes did not have direct influence in increasing periimplantitis risk.
39 [58]	Mo Y.Y. et al.	Medicine	2016	Systematic Review	TNFα (308) gene polymorphism is associated with higher risk of peri-implantitis. The risk is of implant failure is further elevated by smoking. IL-1α (−889) and IL-1β (+3954) are potentially seen as risk factors in peri-implantitis.
40 [59]	Hamdy A.A. et al.	Journal of Oral Implantology	2011	Article	Combination of IL-1α (−889) and Il-1β (+3954) polymorphisms may act as risk factor for tissue destruction and peri-implantitis. Furthermore, IL-1 gene polymorphisms can have negative effect on treatment response and result in genotype-positive patients.
41 [60]	Lafuente-Ibáñez de Mendoza I. et al.	International Journal of Implant Dentistry	2022	Review	There is no direct evidence that IL-1β (+3954), IL-10 (−1081), IL-6 (−174) or TNFα (308) have higher risk of developing peri-implantitis, however composite genotypes IL-1α(−889)/IL-1β (+3954) IL-1α(−889)/IL-1β (+3953) combined with smoking >20 cigarettes a day significantly increase the bone loss and contributes to peri-implantitis.
42 [61]	Laine M.L. et al.	Clinical Oral Implants Research	2006	Article	IL-1β (+3954), IL-1α (−889), IL-1RN polymorphisms were detected more often in peri-implantitis patients and can be assumed to represent as risk factors for peri-implantitis. Smoking increases the chance of peri-implantitis.
43 [62]	Jamshidy L. et al.	International Journal of Environmental Research and Public Health	2021	Review	TNFα (308) gene polymorphism is associated with higher risk of peri-implantitis, especially in Asian ethnicity population
44 [63]	Schminke B. et al.	Journal of Dental Resarch	2015	Research Report	In periimplantitis IL-8, MMP-8 and -9 are upregulated, BMP-9 and PPARγ are downregulated, RUNX levels decrease with the bone destruction progression
45 [64]	Kadkhodazadeh M. et al.	Acta Medica Iranica	2013	Report	IL-17RA polymorphism doesn't increase the chance of periimplantitis incidence
46 [65]	Ingendoh-Tsakmakidis A. et al.	Cellular Microbiology	2019	Research Article	In peri-implantitis, upregulated genes were related either linked to cell division (FIGN, HMGA2, CDC25A, and ERCC6L) or to DNA repair/damage (CLSPN, POLQ, and FANCA). The pathway analysis of the downregulated genes related to this signal transduction (MDM2, IL2RG, TLR4, and F2R). *S. oralis* modulated response of tissues in a way to modulate the peri-implant tissue process.
47 [66]	Mijiritsky E. et al.	Journal of Clinical Medicine	2020	Article	Connective tissue in peri-implantitis has a large number of neutrophils and the structure of the tissue is disorganized, collagen type III production is increased, osteogenic pathways were downregulated, ROS pathways were upregulated
48 [67]	Li Y. et al.	BMC Medical Genetics	2020	Research Article	GSK3B and miR-1297 may have important significance in the immune microenvironment and pathogenesis of peri-implantitis.
49 [68]	Yu T. et al.	PeerJ	2019	Article	There are 92 common genes between periimplantitis and type 2 diabetes, 3 of which (IL6, NFKB1, PIK3CG) are the same, IL-17 expression
50 [69]	Dionigi C. et al.	Journal of Clinical Periodontology	2020	Article	Compared to periodontitis, periimplantitis has increased neutrophil, macrophage and iNOS-positive cells. Epithelium and connective tissue were thicker in periimplantitis than periodontitis.
51 [70]	Li Y. et al.	Frontiers in Immunology	2021	Review	Inflammasomes can be a target point for drugs, periimplantitis tissues have elevated IL-1β and IL-18. Increased NLRP3 levels if *P. gingivalis* or *C. albicans* are present
52 [71]	Galarraga-Vinuenza M. et al.	Clinical Oral Investigations	2021	Article	Enhanced M1 macrophages polarization and higher M1/M2 ratio are found in peri-implantitis sites. M1 phenotype leads to exacerbated osteolysis and inflammatory response, accelerating the peri-implantits progression.
53 [72]	Tzah-Nahman R. et al.	Scientific Reports	2017	Article	Increased PDLF levels stimulate TNFα and IL-1β production as well as spontaneous production of IL-6 by macrophages. Blocking IL-6 or IL-10 production reduced fibroblasts modulatory effect and promoted macrophage phagocytosis when bacterial challenge occurred
54 [73]	Aleksandrowicz P. et al.	Medicators of Infammation	2017	Research Article	Monitoring of MMP-8 level in PISF could help to diagnose mucositis/peri-implantitis in an early stage, prior to clinical manifestations, which may allow for quick start of appropriate therapy.
55 [74]	Guarneri R. et al.	Journal of Personalized Medicine	2022	Article	The initial high level of aMMP8 can be considered as indicators of the subsequent progression of peri-implant bone loss. MMP-8 could be used as biomarker for identifying implants and patients that could present a high bone loss

**Table 2:** Healthy peri-implant vs. peri-implantitis according to 2017 World Workshop on the classification of periodontal and peri-implant diseases and conditions.

Peri-Implant Health	Peri-Implantitis	Peri-Implantitis in the Absence of Previous Examinations
No clinical sign of inflammation	No sign or visible inflammation	No sign or visible inflammation
No bleeding/suppuration on gentle probing	Bleeding/suppuration on gentle probing	Bleeding/suppuration on gentle probing
Stable probing depth between examinations	Increased probing depth compared to previous examinations	Probing depth ≥ 6 mm
No crestal bone changes apart from initial bone remodeling	Crestal bone loss other than initial bone remodeling	Bone levels ≥ 3 mm apical of the most coronal portion of the intraosseous part of the implant

**Table 3:** Pro- and anti-inflammatory cytokines.

Pro-Inflammatory Cytokines	Anti-Inflammatory Cytokines
Interleukin-6	Interleukin-10
Interleukin-1	Tissue Metalloproteinase Inhibitors (TIMPs)
Tumor Necrosis Factor α	Osteoprotegrin
Interleukin-8	Interleukin-1RN
Interleukin-17	Serase Protease Inhibitors (SERPINs)
Metalloproteinase-8 (MMP-8) and other MMPs	

**Table 4:** Molecular factors in peri-implantitis and their function [84,85,86,87,88,89,90,91].

Cytokine	Function
Interleukin-6	Stimulating acute phase protein synthesis, neutrophils production, fever mediation, B-cell growth stimulation
Interleukin-1α	Part of epithelial barrier, epithelium integrity preservation
Interleukin-1β	Modulating inflammatory response, pyrogen, pain hypersensitivity, cell proliferation
Tumor Necrosis Factor α	Immune cells modulation, cell signaling, inflammation regulation, response to bacterial lipopolysaccharide
Interleukin-8	Neutrophil chemotaxis, phagocytosis stimulation
Interleukin-17	Recruitment of immune cells (mainly neutrophils and monocytes) via chemokines, promotes inflammatory responses of IL-1β and TNF-α
Interleukin-10	Anti-inflammatory agent, blocks NFkB activity resulting in decrease in osteoclasts formation, TNF-α regulation
MMP-8	Catalyzes the degradation of collagen type III and I
MMP-2	Collagen type IV degradation, cell-cell clustering
MMP-9	Collagen type IV and V degradation, cooperation with MMP-2 in ECM remodeling
MMP-7	Gelatin, fibronectin and proteoglycan degradation, probably plays role in wound healing
MMP-13	Collagen type I, II and III degradation, tissue remodeling
TIMP-1	MMPs inhibition, cell proliferation promotion
TIMP-2	MMPs inhibition, complements TIMP-1 in maintaining tissues hemostasis
RANKL	Bone remodeling and regeneration control, cell proliferation, with RANK binding promotes osteoclasts formation and maturation
Osteoprotegrin	Suppression of osteoclasts formation by competitive binding to RANK

**Table 5:** Cells present in peri-implantitis tissues and their functions.

Cells Type	Function and Disfunction
Epithelial cells	Apical proliferation, γ-H2AX, iNOS, NOX2, MPO expression
Fibroblasts	Lowered collagen production, mainly type I and III
Macrophages	Tissue infiltration, cytokine production, phagocytosis
Neutrophyls	Tissue infiltration, cytokine production, NETosis, ROS production
Osteocytes	Bone matrix production reduction, inability to repair the damages
Osteoclasts	Bone destruction, influence the bone metabolism
Plasma cells	Maintaining inflammation process, humoral immunity
T-type lymphocytes	Maintaining inflammation process, cellular immunity
Dendritic cells	Inflammation modulation, affect Langerhans cells response

## Data Availability

Not applicable.
